# Thermal Conductivity of Solid Triphenyl Phosphite

**DOI:** 10.3390/molecules27238399

**Published:** 2022-12-01

**Authors:** Alexander Krivchikov, Ove Andersson, Oksana Korolyuk, Oleksii Kryvchikov

**Affiliations:** 1 B. Verkin Institute for Low-Temperature Physics and Engineering of the National Academy of Sciences of Ukraine, 47 Nauky Avenue, 61103 Kharkiv, Ukraine; 2 Department of Physics, Umeå University, 901 87 Umeå, Sweden; 3 Donostia International Physics Center (DIPC), Paseo Manuel de Lardizabal 4, 20018 Donostia-San Sebastian, Spain

**Keywords:** thermal conductivity, triphenyl phosphite, polymorphism, polyamorphism, glass, glacial state, phase transformations, crystals, mechanisms of heat transfer, pressure–temperature diagram

## Abstract

The thermal conductivity, *κ*, of solid triphenyl phosphite was measured by using the transient hot-wire method, and its temperature and pressure dependencies were analyzed to understand heat transfer processes in the solid polymorphic phases, as well as in the glass and the exotic glacial state. Phase transformations and the structural order of the phases are discussed, and a transitional pressure–temperature diagram of triphenyl phosphite is presented. The thermal conductivity of both the crystalline and disordered states is described within the theory of two-channel heat transfer by phonons and diffusons in dielectric solids. In the glass and glacial states, the weakly temperature-dependent (glass-like) *κ* is described well by the term associated with heat conduction of diffusons only, and it can be represented by an Arrhenius-type function. In the crystal phases, the strongly temperature-dependent (crystal-like) *κ* associated with heat transfer by phonons is weakened by significant heat transfer by diffusons, and the extent of the two contributions is reflected in the temperature dependence of *κ*. We find that the contribution of diffusons in the crystal phases depends on pressure in the same way as that in amorphous states, thus indicating that the same mechanism is responsible for this channel of heat transfer in crystals and amorphous states.

## 1. Introduction

Thermal conductivity, *κ*, is a property that is very sensitive to the structure of a substance, making it so that phase transitions can be easily detected, and the structural order of phases can be qualitatively determined by *κ* measurements. It is known that the *κ* of monatomic and simple molecular orientationally ordered crystals normally shows a maximum at low temperatures and then decreases strongly with increasing temperature [1]. The value of *κ* can vary by several orders of magnitude depending on the temperature, and the dependence is determined by the processes of phonon scattering. At temperatures, *T*, well above the thermal conductivity maximum, *κ*, of well-ordered crystals typically decreases with a dependence close to ~1/*T* (Eucken law), which is therefore associated with the crystal-like behavior of *κ*. This temperature dependence is due to phonon–phonon (three-phonon) scattering processes. In complex orientationally ordered molecular crystals, with several molecules in the unit cell, *κ* typically deviates from the Eucken law in the “high-temperature region”. In these cases, *κ* can be approximately represented by a sum of two independent contributions: the first term is the contribution of phonons scattered in phonon–phonon scattering processes (*A/T*, where *A* is a constant), and the second term is a constant contribution (*B*), i.e., a temperature-independent contribution, which takes into account the weaker temperature dependence of *κ* in complex crystals. It is associated with heat transport mediated by vibrations other than those described by (propagating) phonons, and these are sometimes termed diffusons [2,3,4,5,6,7,8,9]. Analyses of experimental results within this theoretical framework suggest that the value of *B* is related to the number of molecules in the unit cell of a crystal [9,10,11].

In amorphous substances, such as glasses and liquids, the *κ* demonstrates a completely different temperature behavior. The characteristic *κ* behavior of glasses, or glass-like behavior, is a low *κ* that increases weakly with temperature [1,12]. The behavior of *κ*(*T*) of complex molecular crystals changes from crystal-like toward glass-like with an increasing degree of structural disorder or increasing number of molecules in the unit cell; in some cases, it fully mimics that of glasses, e.g., in the case of inclusion compounds such as clathrate hydrates. Glass-like behavior of *κ*(*T*) in crystals has been known for decades, but none of the numerous attempts to provide an explanation has found general acceptance. The recent advances in the theory for complex crystals and glasses may provide an explanation, but further evaluation is required. It is therefore of interest to study and analyze the behavior of *κ* and the mechanisms of heat transfer in complex crystals.

Triphenyl phosphite (P(OC_6_H_5_)_3_, TPP) is an intriguing glass-former which has been actively studied for two decades; it exhibits the phenomenon of polymorphism and, perhaps, polyamorphism. The molecule of TPP has a branched shape and can exist in five stable conformations [13]. Over 20 years ago, Ha et al. [14] reported an exotic amorphous state in TPP which they termed a glacial state. They produced the glacial state by heating the glass state formed by rapid cooling of the liquid, which suggests that TPP can exist in two distinct amorphous states. Since then, many studies have focused on establishing the nature of the glacial state (amorphous or nanocrystalline) and the possibility that TPP might exist in two distinct liquid phases, with one of these associated with the glacial state. Studies of the phenomenon include measurements by adiabatic and differential scanning calorimetry (DSC) [15,16,17,18,19,20,21], nuclear magnetic resonance [18,22,23,24], broadband dielectric spectroscopy [20,25], fixed frequency dielectrometry [19], X-ray diffraction [16,22,26,27], Raman scattering [20,26,28], thermal conductivity [17], and neutron diffraction [29]. A review by Tanaka [30] discusses the possible existence of two or more liquids (and glasses) for a single-component substance, in particular for TPP. As discussed previously, there are conflicting views on the nature of the glacial state [17]. In a paper by Hédoux et al. [31], the authors analyze the transformation into the glacial state, which is interpreted as a heavily nucleated state composed of nanocrystals of the stable crystalline phase embedded in a matrix of non-transformed supercooled liquid. Demirjian et al. [22] investigated TPP up to pressures of 0.6 GPa and have shown that the glacial phase is well described as a plastic crystal composed of nano-crystallites. The results in a previous study of the thermal conductivity and heat capacity of TPP [17] show that the sluggish liquid-to-glacial-state transformation occurs at temperatures about 15 K above the glass transition temperature, *T*_g_, and suggest that the final state is a heterogeneous mixture of nanocrystals and a mostly amorphous solid.

The crystallization behavior of TPP was recently investigated by DSC and infrared spectroscopy [32,33]; the results show that TPP can crystallize in two different phases at atmospheric pressure: a stable phase and a metastable phase. DSC runs on heating show that the metastable crystal melts at ∼291.6 K, while the melting temperature of the stable phase is ∼299.1 K [32].

Here we present a comprehensive study and analysis of *κ* of the various phases and states of TPP under pressure, *P*; it supplements a previous study of *κ* during the supercooled-liquid-to-glacial-state transformation [17]. In particular, we report the *κ*(*T*, *P*) behavior and the mechanisms of heat transfer in the solid TPP phases, including the stable and metastable crystalline phases, the glass, and the exotic glacial state. The analysis in terms of two-channel heat transport provides an important extension—the pressure dependence of *κ*—compared to previous studies. Moreover, phase transformations and the structural order of the phases are discussed, and a transitional pressure–temperature diagram of TPP is provided.

## 2. Results

The glass state of the sample was produced by cooling the liquid at a rate of 1.5–2.0 K/min to a temperature below the glass transition temperature, *T*_g_; *κ* results of the glass state are summarized in Section 2.1, in the Results.

The glacial state was produced by heating the glass state to a temperature slightly above *T*_g_ and waiting for a complete transformation of the supercooled liquid (SCL) at constant conditions: 220 K at 0.00 GPa, 227 K at 0.05 GPa, 237 K at 0.1 GPa, and 296 K at 0.48 GPa. The glacial state was also produced by pressuring the liquid up to *P* = 0.48 GPa at *T* = 330 K. At the transition, *κ* increases weakly by Δ*κ* ≈ 0.007 W m^−1^ K^−1^, see details in Ref. [17]. When the glacial state is heated, the sample crystallizes into a metastable crystalline state, which we have designated as crystal*II, at the transition temperature T*; *κ* results of the glacial state are presented in Section 2.2.

The temperature (and pressure) dependence and size of *κ* in polycrystalline phases depend on both the crystalline structure and the quality of the crystals, i.e., crystal sizes and disorder such as dislocations and stacking faults. Here we use *κ*(*T*) to identify two distinct (poly)crystalline phases, crystal I and crystal II, as well as a relatively stable pre-state of crystal II with significant disorder (crystal*II). Crystal I is a stable equilibrium polymorph with a high *κ* that strongly increases with decreasing *T*. Crystal II is a metastable polymorph that is easily distinguished from crystal I by its different *κ* characteristics, specifically the magnitude and temperature dependence of *κ* (see Section 2.3). Based on the transition and *κ* behaviors, we assert that crystal*II is the same polymorph as crystal II, but with many crystal defects that reduce the magnitude and temperature dependence of *κ*. (XRD studies were not carried out.) The phases were produced by several different *P*–*T* paths. Crystal*II was obtained by (i) liquid–solid transformation by heating from SCL, bypassing the sluggish SCL-glacial transformation, (ii) solid–solid transformation by heating from the glacial state, and (iii) liquid–solid transformation by compressing the SCL at quasi-isothermal conditions. In all of these cases, the transformation of the sample occurred while passing through the transition temperature, T*, without annealing; the *κ* of crystal*II shows only a weak temperature dependence (see Section 2.3). A sample that was annealed or slowly heated near T* showed gradually, and irreversibly, increasing values of *κ* with time due to an improved degree of crystallinity. This shows that phase II* is not a well-defined state, yet the changes are sufficiently small so that it is relevant to describe the general properties of phase II*. Upon further heating above T*, crystal*II transforms irreversibly and distinctly into crystal II. Polycrystalline samples of crystal II were also obtained at atmospheric pressure by cooling the liquid from 300 K to *T* ≈ 248 K and subsequent heating to *T* ≈ 270 K. When crystal II is heated above the transition temperature *T*_I_ and annealed near the melting point under isobaric condition, it transforms irreversibly into crystal I. The stable polymorph crystal I was also obtained by liquid–solid transformation by cooling the liquid state and annealing just below the melting point at isobaric conditions.

### 2.1. The Thermal Conductivity of the Glass State

**Figure 1:**
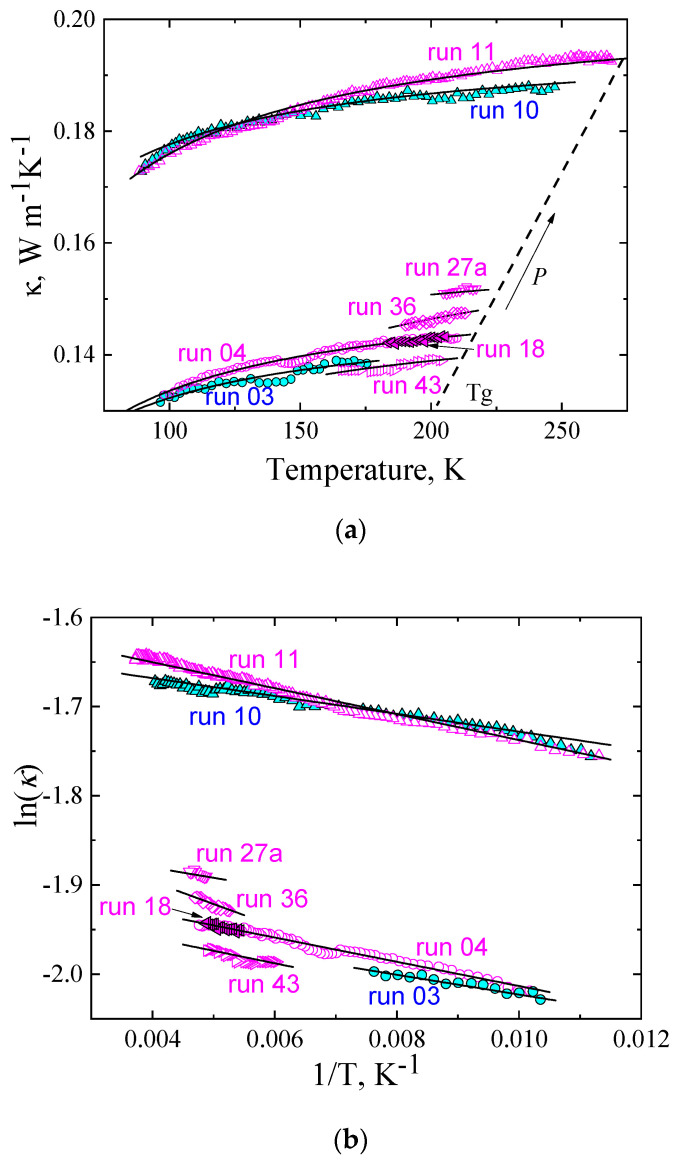
Thermal conductivity of TPP in glass state: (**a**) thermal conductivity plotted against temperature; (**b**) natural logarithm of thermal conductivity plotted against inverse temperature. Cyan symbols are for cooling, and magenta symbols are for heating. Black solid lines represent fitted functions of Equation (1). The dashed line shows the change of the glass transition temperature, *T*_g_, with pressure.

The results in Figure 1a show the isobaric *κ*(*T*) of TPP glasses. Cyan symbols are for cooling, and magenta symbols are for heating. The results differ slightly between heating and cooling, and this is due to slightly different pressures caused by the reversal of frictional forces [17] and temperature gradients caused by high cooling rates. The values of *κ* of the glass are low and only weakly temperature dependent, as is consistent with the general behavior of glasses and previously reported results for TPP [17]. For example, at 0.05 GPa, *κ* increases only 7.5% at a temperature increase of 110 K (run 04); likewise, at 0.48 GPa, *κ* increases 9.2% at a temperature increase of 150 K (run 11). The pressure variation of *κ* (=Δ ln *κ*/Δ*P*), calculated from the isobaric data at 150 K (Figure 1a), is 65% GPa^−1^, which is a typical variation for glasses; for example, it is the same as that for the glassy polymer poly(methyl methacrylate) [34]. The dashed line in Figure 1a shows the increase of the glass transition temperature, *T*_g_, with increasing pressure.

The isobaric temperature behavior of the *κ* of glasses is well described by an Arrhenius-type function:

*κ*(*T*, *P*) = *κ*_0_(*P*) exp(−*E*/*T*), (1)

where *E* is the activation energy expressed in K, and *κ*_0_ is a pre-exponential factor; it corresponds to the maximum (limiting) *κ* that the state attains at high temperatures. The solid lines in Figure 1a show the fitted Arrhenius functions, *κ*(*T*, *P*); the fitted functions and the experimental results agree within about 1%.

In order to determine the values of *E* and *κ*_0_, we have ln(*κ*) versus 1/*T* in Figure 1b (same symbols as in Figure 1a). The experimental data are well described by straight lines with the slope equal to −*E* (Equation (1)). The values of the two fitting parameters, the pre-exponential factor, *κ*_0_, and activation energy, *E*, are summarized in Table 1; *E* shows no systematic pressure variation, whereas, as expected, *κ*_0_ increases with increasing pressure.

**Figure 2:**
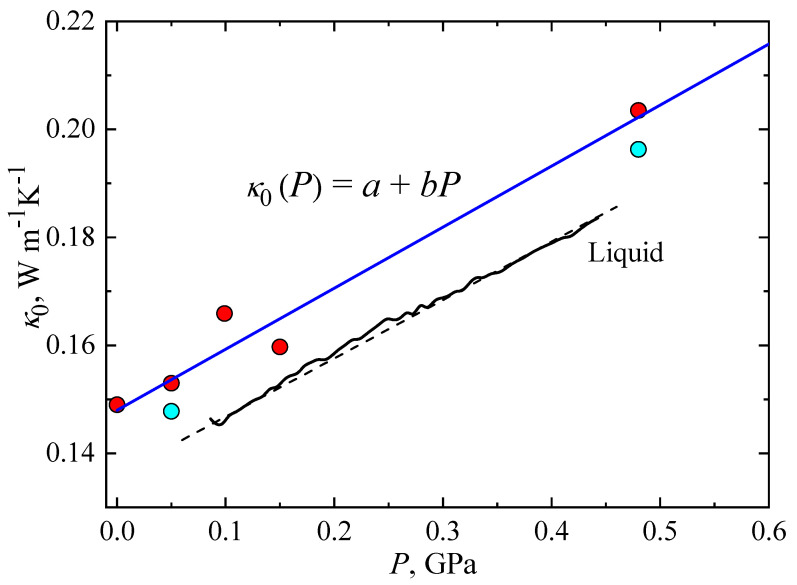
Pre-exponential factor, *κ*_0_, of the glass state plotted against pressure. Red and cyan symbols correspond to results measured on heating and cooling, respectively. The blue line represents a fit of Equation (2). The black solid line shows *κ*(*P*) for the liquid at 332 K; the dashed black line represents a linear fit: *κ*(*P*) = 0.136 Wm^−1^K^−1^ + 0.108 P Wm^−1^K^−1^GPa^−1^.

Figure 2 shows the pre-exponential factor, *κ*_0_, as a function of pressure for TPP glass. Red symbols correspond to heating and cyan symbols correspond to cooling; see Table 1 for details. The results show that κ_0_ varies linearly with pressure:

*κ*_0_(*P*) = *a* + *bP*, (2)

where *a* = 0.148 Wm^−1^K^−1^, and *b* = 0.113 Wm^−1^K^−1^GPa^−1^ is the rate of change of *κ*_0_(*P*) with pressure. The value of *b* is close to the value obtained for the isothermal pressure dependence of *κ* of the glass state: (Δ*κ*/Δ*P*)*_T_* = 0.108 Wm^−1^K^−1^GPa^−1^, which was reported in a previous study of TPP [17]. For comparison, in Figure 2, we show the κ of liquid TPP as a function of pressure (solid black line, *T* = 332 K) and a fitted function: *κ*(*P*) = 0.136 Wm^−1^K^−1^ + 0.108 *P* Wm^−1^K^−1^GPa^−1^ (dashed black line). As shown, the pressure variations of *κ*_0_ of the glass (blue line) and the liquid (dashed black line) are almost identical; this is a consequence of the close structural relationship between the two states and shows that the kinetic unfreezing of molecular motions at the glass–liquid transition does not significantly affect the pressure dependence of *κ*.

### 2.2. The Thermal Conductivity of the Glacial State

**Figure 3:**
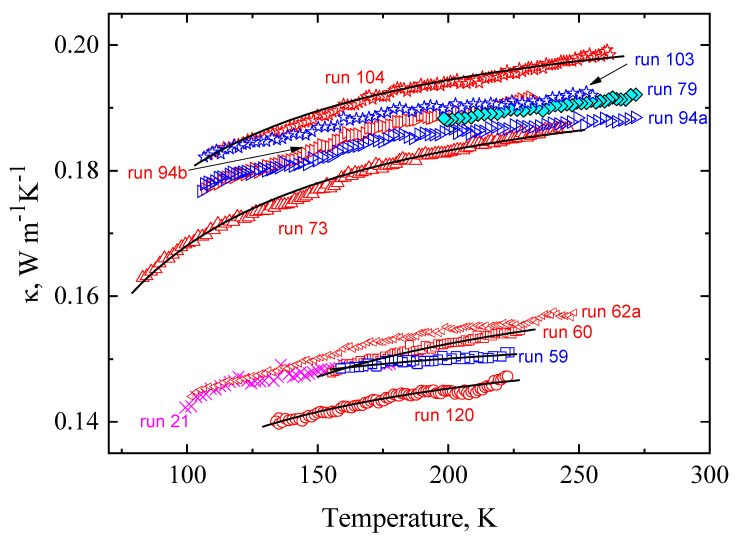
Thermal conductivity of TPP in the glacial state plotted against temperature. Blue and cyan symbols are for cooling. Red and magenta symbols are for heating. Black solid lines represent fitted functions of Equation (1).

The isobaric *κ*(*T*) values of the glacial state of TPP are shown in Figure 3; blue and cyan symbols are for cooling, and red and magenta symbols are for heating. (As discussed in Section 2.1, the results differ slightly between measurements on heating and cooling.) The thermal conductivity of the glacial state is low, about the same as that of the glass; as in the glass, the *κ* increases only weakly with temperature. For example, at 0.49 GPa, *κ* increases only 8.1% at a temperature increase of 150 K (run 104). Upon isothermal pressurization at 150 K, *κ* increases 47% GPa^−1^, as derived from the isobaric data at 0.05 and 0.5 GPa in Figure 3.

**Figure 4:**
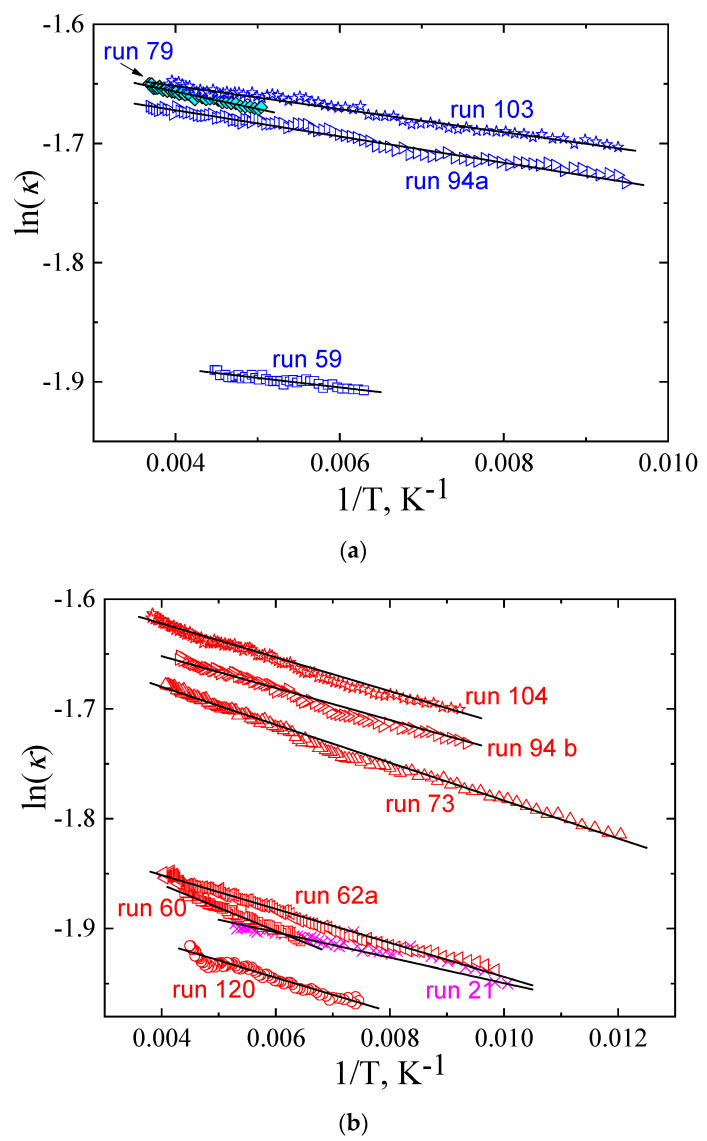
Thermal conductivity of TPP in the glacial state: (**a**) natural logarithm of thermal conductivity measured upon cooling is plotted against inverse temperature; (**b**) natural logarithm of thermal conductivity measured upon heating is plotted against inverse temperature. Black solid lines represent fitted functions of Equation (1).

The temperature behavior of *κ* of the glacial state is similar to that of the glass, and the same function, Equation (1), was used to model the data. The solid lines in Figure 3 represent fits of Equation (1) to data for the *κ*(*T*) at 0.0 GPa, 0.10 GPa, 0.49 GPa, and 0.5 GPa. The values for *E* and *κ*_0_ were determined by the same procedure as that used for the glass, i.e., in plots of ln (*κ*) versus 1/*T*; as depicted in Figure 4, the linear variation of the plots, with the slope equal to *−E*, shows that Equation (1) also provides a good description of the *κ*(*T*) of the glacial state. Table 2 lists the two fitting parameters, *E* and *κ*_0_, for the *κ*(*T*) of the glacial state.

**Figure 5:**
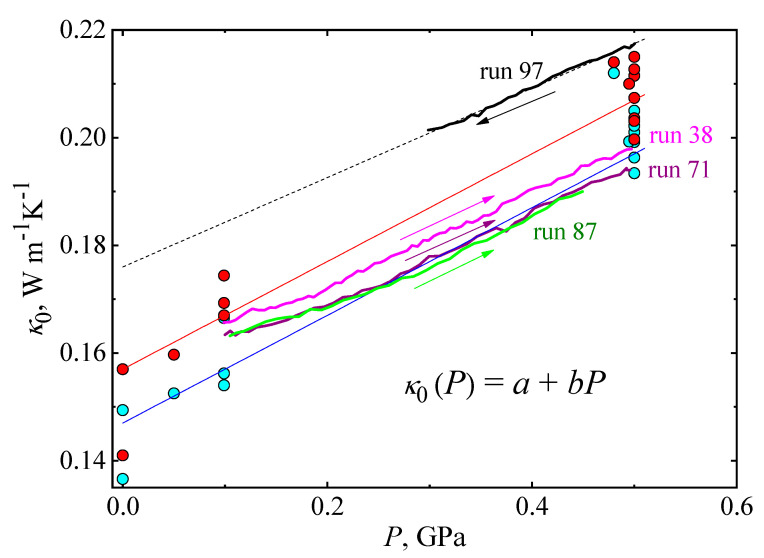
Pre-exponential factor *κ*_0_ of the glacial state plotted against pressure. Red and cyan symbols correspond to results measured upon heating and cooling, respectively (see Table 2). The red and blue straight lines represent the fits of Equation (2). Magenta, purple, green, and black lines show *κ*_0_(*P*) calculated from the experimental values of *κ*(*T*, *P*) via Equation (1), with *E* = 15 K (for runs 38, 71, 87, and 97). The arrows indicate the direction of pressure change. The black straight dashed line is a fit of Equation (2), with parameters *a* = 0.176 Wm^−1^K^−1^ and *b* = 0.083 Wm^−1^K^−1^GPa^−1^ (for run 97).

As in the case of the glass state, the value of the pre-exponential factor, *κ*_0_, increases with increasing pressure. Figure 5 shows the pressure variation of *κ*_0_ of the glacial state. Red symbols correspond to the results upon heating, and cyan symbols correspond to those upon cooling (see Table 2). The larger scatter in the *κ*_0_ of the glacial state compared to that of the glass state (see Figure 2) suggests that the *κ* of the glacial state is more thermal-history-dependent than the *κ* of the glass [17]. As depicted in Figure 5, *κ*_0_ changes about linearly with pressure and the fits of Equation (2) yield: *a* = 0.157 Wm^−1^K^−1^, *b* = 0.1 Wm^−1^K^−1^GPa^−1^ (red line, heating), and *a* = 0.147 Wm^−1^K^−1^, *b* = 0.1 Wm^−1^K^−1^GPa^−1^ (blue line, cooling). As found for the glass state, the value of *b* is close to the value reported previously for the pressure dependence of *κ*, (Δ*κ*/Δ*P*)*_T_* = 0.114 Wm^−1^K^−1^GPa^−1^ [17].

For a direct comparison between the *κ*_0_(*P*) derived from isobaric measurements and values derived from isothermal measurements, we calculated the *κ*_0_ from datasets of *κ*(*P*) at constant temperatures. Direct measurements of *κ*(*P*) of the glacial state were carried out by pressurization at 237 K and depressurization at 295 K; Equation (1), with the average value of *E* (=15 K), yields the *κ*_0_(*P*), and the results are depicted in Figure 5. A fit of Equation (2) to the dataset at 295 K yields *a* = 0.176 Wm^−1^K^−1^ and *b* = 0.083 Wm^−1^K^−1^GPa^−1^ (black dashed line). As shown in Figure 5, the pressure dependence is essentially the same for the different datasets, whereas the magnitudes differ slightly. The latter suggests that the magnitude of the *κ*_0_ of the glacial state depends slightly on the thermal history.

Comparing the *κ*(*T*, *P*) values of the glass and glacial states of TPP, it is obvious that the behavior is remarkably similar with low values for *κ*, which increases only weakly with increasing temperature and pressure. This similarity, which suggests a structural relationship between the glass and glacial states, is demonstrated quantitatively by the similar values of the parameters of Equations (1) and (2), i.e., *E*, *κ*_0_, *a*, and *b*. We note, however, that although the *κ* of a glass is known to be dependent on thermal history, this dependence is smaller than that for the glacial state [17].

### 2.3. The Thermal Conductivity of Crystal I, Crystal II, and Crystal*II

**Figure 6:**
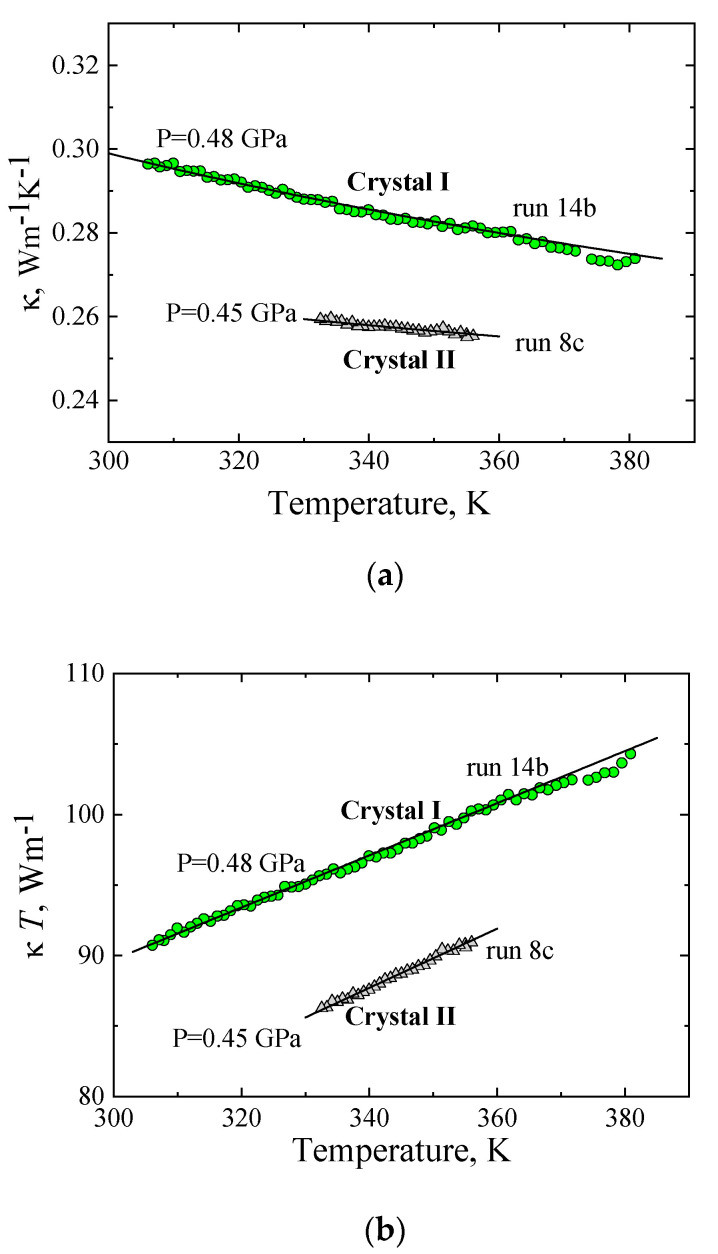
Temperature behavior of thermal conductivity of the stable crystal I and metastable crystal II phases: (**a**) Thermal conductivity of TPP plotted against temperature: crystal I (green circles, run 14b) and crystal II (gray triangles, run 8c). Solid lines represent fits of Equation (3). (**b**) The product *κ*(*T*)·*T* plotted against temperature: crystal I (green circles, run 14b) and crystal II (gray triangles, run 8c). Parameters *A* and *B* in Equation (3) are determined by straight line fits: *κ*(*T*)·*T* = *A* + *B* ·*T* (black solid lines).

The temperature behavior of the κ of crystals in the region where phonon–phonon scattering processes predominate is depicted in Figure 6. Figure 6a shows the *κ*(*T*) of the stable crystal I phase of TPP at 0.48 GPa (run 14b) and *κ*(*T*) of the metastable crystal II phase at 0.45 GPa (run 8c). At these relatively high temperatures, phonon–phonon scattering is the dominant phonon-scattering mechanism in crystalline phases. As a consequence, both the magnitude of the κ and its temperature dependence differ radically from the *κ*(*T*) of the glass and glacial states (see Figure 1a and Figure 3). In particular, in this temperature range, the κ of crystals decreases with increasing temperature. We also note that the κ of crystal I is about 10% higher at 340 K and more dependent on temperature than that of crystal II. These features are indicative of higher crystalline order or fewer molecules per unit cell in crystal I than in crystal II.

In the temperature range where phonon–phonon-scattering processes predominate, the *κ*(*T*) of molecular crystals follows a model that includes two additive independent contributions (see, for example, refs. [2,3,4,5,6,7,8,9]. In this model, *κ*(*T*) can be expressed as follows:

*κ*(*T*) = *A*/*T* + *B*, (3)

where *A*/*T* is the contribution from phonons (propagating phonons), which are scattered in phonon–phonon processes, with *A* being a parameter that depends on, for example, lattice anharmonicity and phonon velocity; the second term, *B*, is a temperature-independent contribution associated with diffusons [3,4,5,35]. The thermal conductivity of all crystalline phases of TPP is well described by Expression (3), as shown by the solid lines in Figure 6a. To determine the contributions from phonons (*A*/*T*) and diffusons (*B*) in Equation (3), *κ*(*T*) ·*T* is plotted against temperature in Figure 6b; the slope determines the contribution of diffusons *B*, and *A* is the intercept. Table 3 shows the *A* and *B* values of crystal I and crystal II at various pressures.

**Figure 7:**
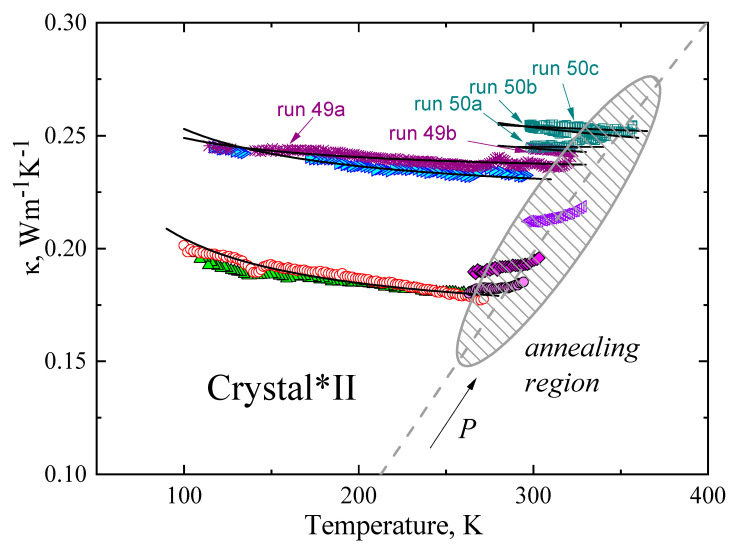
Thermal conductivity of crystal*II plotted against temperature at 0.05 GPa run 05 (▲) and run 06a (○); 0.10 GPa run 62b (●); 0.15 GPa run 27b (♦); 0.3 GPa run 32 (◁); 0.47 GPa run 48 (►), run 49a,b (✳), and run 50a,b,c (□). Black solid lines represent fits of Equation (3), with parameters *A* and *B*, which are listed in Table 3. The arrow indicates the direction of increasing pressure and temperature of the sluggish ordering transformation of crystal *II (gray dash line). The shaded area indicates the annealing region.

Figure 7 depicts the *κ*(*T*) of crystal*II of TPP at various pressures, and the black solid lines are the fits of Equation (3). The fitting parameters (*A* and *B*) were determined in the same way as for crystals I and II and are listed in Table 3. The good agreement between the fitted lines and the experimental data shows that the model (Equation (3)) provides a good description of the *κ*(*T*), at least up to the range indicated by the shaded zone in Figure 7, which we refer to as the annealing region.

In the low-temperature range of the shaded zone, the *κ* of TPP remains almost constant in a narrow range of about 17 K (see runs 62b, 27b, 32, and 50a); however, upon further heating toward the crystal*II to crystal II transition temperature, *T*_II_, we note an annealing effect in the *κ*(*T*) of crystal*II. This is manifested by an irreversible increase of the *κ* upon heating, where the irreversibility is observed through a slight time-dependence in *κ*, as well as a higher *κ* upon subsequent cooling than upon first heating. This is demonstrated by temperature cycling at 0.48 GPa: first heating, run 49a → subsequent cooling, run 49b; second heating, run 50a → second cooling, run 50b → third heating, run 50c. The irreversibility is likely due to sluggish growth and reformation of crystals that increase the crystal quality on annealing to temperatures in the upper range of the shaded zone in Figure 7.

The results in Figure 7 also show that the *κ* increases with pressure. The arrow in Figure 7 indicates the direction of the increasing pressure. For example, at *T* = 200 K, the *κ* of TPP at 0.48 GPa (run 49a) is 1.3 times higher than that at 0.05 GPa (run 05).

Table 3 shows the results of fitting Equation (3) to the *κ*(*T*) of the crystalline phases I, II, and *II; the three phases can be clearly distinguished because of their different *κ*(*T*) behavior, which is quantified in the parameter values of *A* and *B*. The stable polymorph crystal I shows the highest value for *A*; the *A* value of metastable polymorph crystal II is about 2–3 times less, and crystal*II shows the lowest *A* value, about 20 times less than that of crystal I. The contribution of diffusons, as specified by *B*, shows the opposite behavior. That is, it is largest in phase *II and least in phase I; the difference is about 30% at atmospheric pressure. These differences typically reflect the effect of a changing crystalline structure.

**Figure 8:**
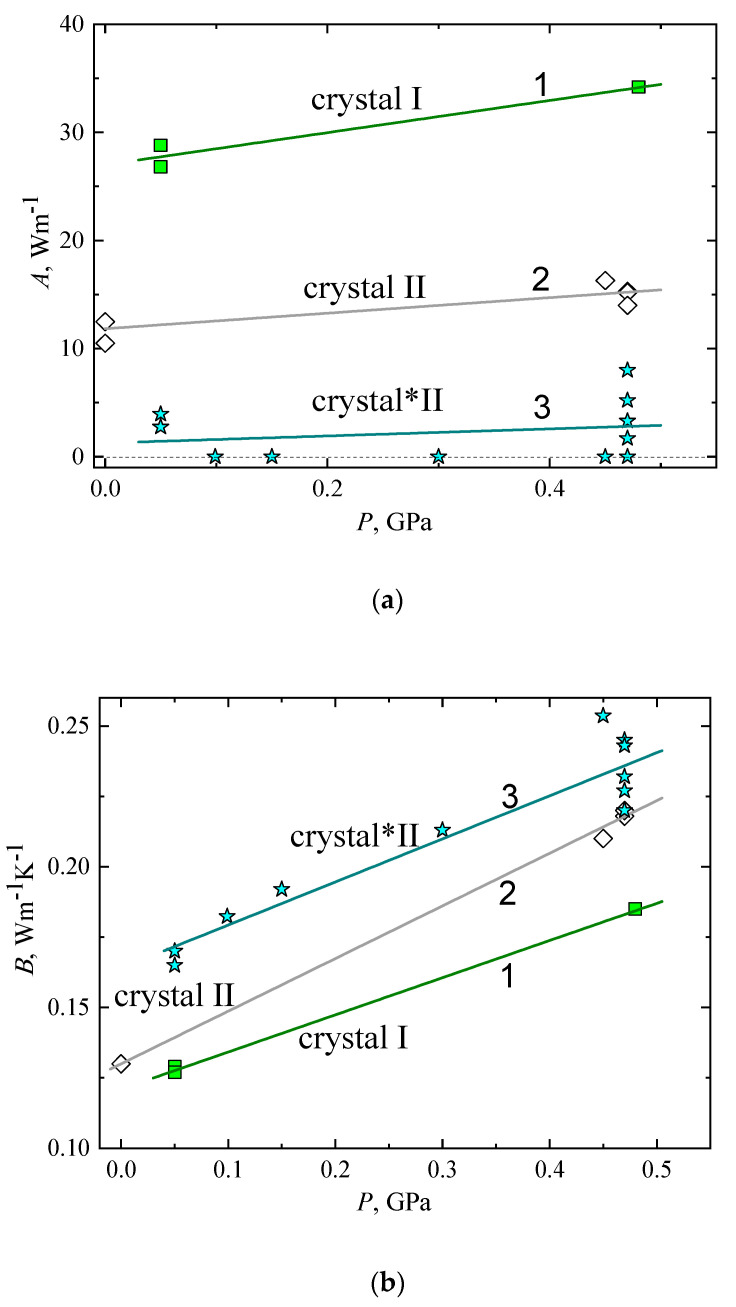
Pressure dependence of parameters *A* and *B* for crystal I, II, and *II: (**a**) Pressure dependence of the parameter *A* for (■) crystal I, (◊) crystal II, and (star) crystal*II. Solid lines (1, 2, and 3) represent fits of Equation (4). (**b**) Thermal conductivity contribution from diffusons, *B*, are plotted against pressure for (■) crystal I, (◊) crystal II, and (star) crystal*II. Solid lines (1, 2, and 3) represent fits of Equation (5).

Figure 8 depicts the pressure dependence of the parameters *A* and *B* in Equation (3) for crystal phases I, II, and *II. Figure 8a shows the pressure dependence of *A*, which is proportional to the pressure dependence of the phonon contribution to the *κ* at constant temperature. Symbols are experimental values, and straight lines (1, 2, and 3) are linear approximations for crystal I, crystal II, and crystal*II, respectively.

The relatively weak pressure dependence of *A* can be described by linear functions and shows that the *κ* associated with phonons increases roughly linearly with pressure; thus, the parameter *A* varies as follows:

*A*(*P*) = *A*_av_ + (*dA*_av_/*dP*)·*P*, (4)

where *A*_av_ is an average value at atmospheric pressure, and *dA*_av_/*dP* is the constant rate of change with pressure. The values of *A*_av_ and *dA*_av_/*dP* for crystal I, crystal II, and crystal*II are shown in Table 4. Crystal I shows the largest increase of *A* with increasing pressure, crystal*II shows the least growth, and crystal II shows an intermediate behavior.

Figure 8b shows the pressure dependence of *B*, which is the diffuson contribution to the *κ*. Experimental values are represented by symbols, straight lines (1, 2, and 3) are linear approximations for the cases of crystal I, crystal II, and crystal*II, respectively. Lines (1, 2, and 3) show that the contribution of diffusons, *B*, increases roughly linearly with a pressure like that for the glass and glacial states:

*B*(*P*) = *B*_av_ + (*dB*_av_/*dP*)·*P*, (5)

where *B*_av_ is an average value at atmospheric pressure, and *dB*_av_/*dP* is the rate of change with pressure. The values of *B*_av_ and *dB*_av_/*dP* for the crystal phases are shown in Table 4, together with the parameters *a* and *b* in Equation (2) for the glass and glacial states.

### 2.4. Phase Transformations

**Figure 9:**
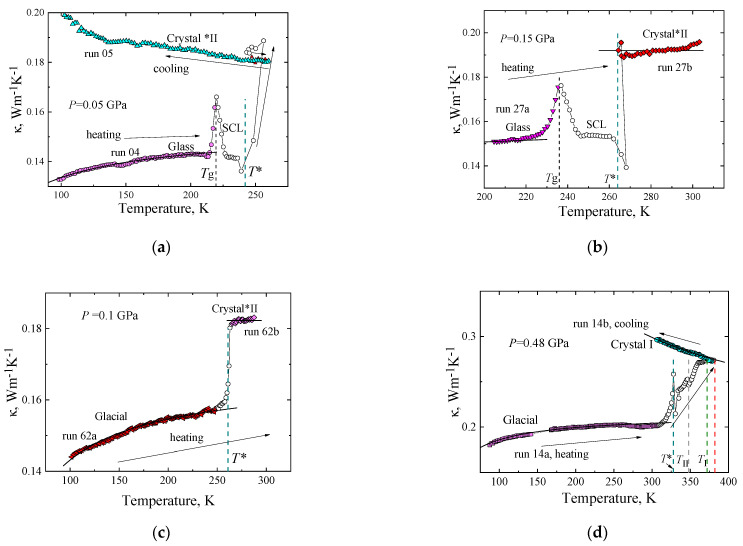
Transformations detected in *κ* of TPP measured at isobaric conditions. Arrows indicate the directions of temperature and *κ* changes. Dashed lines correspond to transition temperatures: glacial → crystal*II at T* (cyan), crystal*II→ crystal II at *T*_II_ (gray), crystal II→ crystal I at *T*_I_ (green). Red dashed line indicates the melting temperature. In panels (**a**–**d**), (○) corresponds to either a transition process or a supercooled liquid state. Solid black lines represent fits of Equation (1) to *κ*(*T*) of the glass and glacial states, and fits of Equation (3) to *κ*(*T*) of the crystalline phases: (**a**) The sample, initially in a glass state (run 04, ●), transforms into the supercooled liquid phase that crystallizes into crystal*II (▲), (▲) *κ*(*T*) of crystal*II measured on subsequent cooling (run 05). Dashed lines correspond to transition temperatures: *T*_g_ glass–liquid (black) and T* supercooled liquid to crystal*II (cyan). (**b**) The glass state (run 27a, ▼) transforms to supercooled liquid at *T*_g_ (black), which crystallizes into crystal*II (run 27b, ♦) at T* (cyan); (c) The glacial state (run 62a, ◄) crystallizes into crystal*II (run 62b, ♦) at T* (cyan); (d) The glacial state (run 14a, ■) crystallizes into crystal I through a series of transitions; (●) *κ*(*T*) of crystal I measured upon cooling (run 14b).

Triphenyl phosphite exists in two different crystalline polymorphous structures and two solid polyamorphous states: a glass state and a glacial state, which are vitrified states of the supercooled liquid phase or, in the two-liquid model, vitrified states of two different liquid phases. The transformations between these were detected during measurements of the *κ*(*T*, *P*) of TPP. Liquid-to-glass transitions occurred upon cooling and pressurization, and glass-to-liquid transitions upon heating and depressurization. The transformation from the (supercooled) liquid to the glacial state was detected upon very slow heating and long-time annealing at temperatures slightly above the glass transition [15,16,31]. Moreover, glacial-to-crystal and liquid-to-crystal transitions, as well as crystal–crystal transitions, were also detected upon further heating. Figure 9 shows typical examples of transition sequences. The arrows indicate the direction of temperature and *κ* change. The transition temperatures are indicated by dashed lines: the transition temperature of glass to (supercooled) liquid, *T*_g_ (black); the crystallization temperature crystal*II, T* (cyan), the transition temperature of crystal*II to crystal II, *T*_II_ (gray); and the transition temperature of crystal II to crystal I *T*_I_ (green). The red line indicates the melting temperature.

Figure 9a shows the *κ* measured upon heating at 0.3 K/min at 0.05 GPa (run 04) after it had been cooled from the liquid phase at 1.9 K/min at 0.05 GPa. The results show that the sample is initially in the glass state and that it passes through the *T*_g_ at about 219 K, which produces an artificial peak in the *κ* due to the time-dependent heat capacity in the glass-to-liquid transition range [36]. On further heating, the supercooled liquid phase crystallizes into crystal*II at T*, and the *κ* increases approximately 1.3 times. With this thermal treatment, i.e., heating at a constant rate of 0.3 K/min, the supercooled-liquid-to-glacial transformation does not occur because of its requirement of a dwell time of about an hour or longer before initiation, and thereafter a transformation process that lasts for several hours. Upon the subsequent cooling of crystal *II at 0.05 GPa (run 05), the *κ* increases with decreasing temperature, in sharp contrast to the decreasing *κ* of the glass state.

The results of *κ*(*T*) measured upon heating at 0.4 K/min rate at 0.15 GPa, which are depicted in Figure 9b, show identical transition behavior as at 0.05 GPa; that is, the glass state of TPP (run 27a) undergoes a glass transition at *T*_g_ prior to crystallization into crystal *II at T* (run 27b). The glass–liquid transition peak in the *κ* and the crystallization-induced increase of the *κ* (Δ*κ* ≈ 0.039 Wm^−1^K^−1^) are, however, shifted to higher temperatures than those at 0.05 GPa.

Figure 9c shows the *κ*(*T*) of the glacial state measured upon heating at 0.10 GPa. (The glacial state was obtained after a long time of annealing of the supercooled liquid at temperatures slightly above *T*_g_ [17].) The results show that the glacial state (run 62a) crystallizes, initially slowly and then more rapidly, into crystal *II at T* (run 62b). During this transition, the *κ* increases by Δ*κ* ≈ 0.023 Wm^−1^K^−1^, and the temperature dependence of the *κ* changes from weakly positive in the glacial state to constant in crystal *II. Since the *κ* of crystal *II increases upon cooling (Figure 9a), it suggests that the microstructure of crystal *II changes gradually, increasing the crystallinity, upon heating. As a consequence, the increase in phonon–phonon scattering upon heating is compensated by a decrease in phonon scattering against structural defects, and the *κ*(*T*) remains constant.

Figure 9d shows the *κ*(*T*) measured upon heating of the glacial state at a higher pressure (run 14a), and the transition behavior differs slightly from that at 0.1 GPa. At 0.48 GPa, the glacial state transforms into crystal I through a series of transitions with no obvious (meta)stable temperature range of the intermediate phases. First, there is a solid–solid transition from the glacial state to crystal*II at temperature T*; then crystal*II transforms into crystal II at *T*_II_; and finally, crystal II transforms into crystal I at *T*_I_. Upon heating and passing through the temperatures T* and *T*_II_, a jump in *κ* occurs. With further heating and passing through the temperature *T*_I_, a kink occurs in the *κ*(*T*) curve. This behavior of the *κ*(*T*) indicates that the transitions at T*, *T*_I_, and *T*_II_ are first-order transitions. As a rule, first-order transitions form a single phase, but not a mixture of two phases. Upon the slow heating of crystal I, annealing occurs with an improvement in the quality of the crystals, and a consequential increase in the *κ* takes place. At each transition, the magnitude of the *κ* increases. The overall change in the *κ* during the transition sequence is large; for example, at 300 K, the *κ* of crystal I is approximately one and a half times greater than that of the glacial state. Moreover, upon a subsequent temperature decrease of the stable equilibrium crystal I (run 14b), the *κ*(*T*) increases, which further distinguishes its *κ* from that of the glacial (and glass) state.

### 2.5. Transitional Pressure–Temperature Diagram

**Figure 10:**
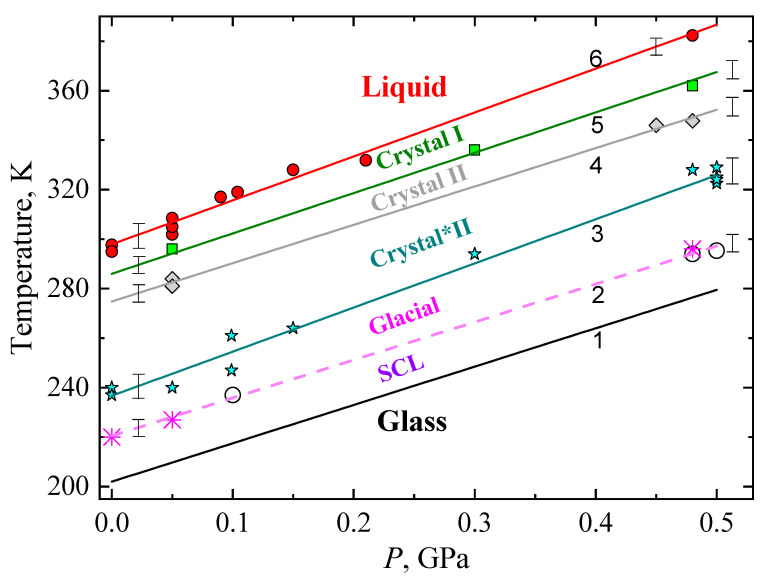
Transitional pressure–temperature diagram of stable and metastable states. Symbols (●, ■, ♦, stars, and *) are experimental data of this work, and symbol (○) refers to experimental data from [17] for the SCL-to-glacial-state transition. Lines: Black line 1 shows *T*_g_ [17]; pink dashed line 2 corresponds to the irreversible transition from the SCL to the glacial state, *T*_gl_(*P*) = 220.5 K + 153.4·*P* K/GPa; dark cyan line 3 corresponds to an irreversible transition from the glacial state to crystal*II, T*(*P*) = 236.7 K + 178.3·*P* K/GPa; gray line 4 corresponds to an irreversible transformation from crystal*II to crystal II, *T*_II_(*P*) = 275 K + 155·*P* K/GPa; olive line 5 corresponds to an irreversible transition from crystal II to crystal I, *T*_I_(*P*) = 286 K + 163·*P* K/GPa; and red line 6 is the melting temperature, *T*_m_(*P*) = 298 K + 178·*P* K/GPa. The vertical bars show the inaccuracy (standard error) in the transition temperatures based on the scatter in the data.

A (P–T) pressure–temperature phase diagram of TPP was constructed based on the phase-transition observations in the *κ*(*T*). Figure 10 shows the (meta)stable ranges of the glass and crystal states and the crystalline phases. Closed symbols are the experimental data of the present work. The straight black line 1 is the *T*_g_(*P*) drawn according to the data in ref. [17], where the glass transition temperature is *T*_g_ = 218 and 276 K at 0.1 and 0.48 GPa, respectively, and (∂*T*_g_/∂*P*) ≈150 K GPa^−1^. The values for *T*_g_ reported here correspond to a structural relaxation time of 0.3 s [36]. In an earlier study, Mizukami et al. [16] reported that T_g_ = (200 ± 2) K from adiabatic calorimetry results (slow heating) at atmospheric pressure (not shown in Figure 10), which is in good agreement with line 1. The values of *T*_g_ measured upon heating are several degrees (≈ 7–8 K) higher than those obtained during cooling; this is mainly due to the difference in pressure caused by the reversal of frictional forces, but also due to changes in the temperature gradients in the sample cell. The *T*_g_ also depends on the heating rate; Wiedersich et al. [18] reported a value of 205 K upon heating at 10 K/min rate at atmospheric pressure. Mierzwa et al. [25] reported that *T*_g_ = 204 K at ambient pressure, and 266 K at 0.5 GPa from broadband dielectric spectroscopy; these results are consistent with our data.

The area bounded by lines 1 and 2 in Figure 10 corresponds to the existence of supercooled liquid. However, because of the sluggish supercooled liquid-to-glacial transformation, and the requirement here of a dwell time at a constant temperature to detect the onset, the coordinates of line 2 are somewhat arbitrary; but the pink dashed line 2 provides a suitable temperature *T*_gl_ for obtaining the glacial state by long-time annealing of the supercooled liquid phase at elevated pressures, and it is given by *T*_gl_(*P*) = 220.5 K + 153.4·*P* K/GPa. For comparison, Wiedersich et al. [18] obtained a transition temperature of 224 K upon heating at a rate of 0.5 K/min at atmospheric pressure, and this is in good agreement with our data despite the differences in experimental conditions. NMR data at 0 GPa and 0.4 GPa show supercooled-liquid–glacial-state transformations at temperatures that are in fair agreement with line 2 [22].

If the supercooled liquid was heated at a constant rate, then the supercooled-liquid-to-glacial transformation was not detected in *κ*(*T*). This can be seen in the examples depicted in Figure 9a (run 04) and Figure 9b (run 27a,b). In these cases, the supercooled liquid was heated at a rate of about 0.3 K/min and 0.4 K/min, respectively; the crystallization of the sample into crystal*II immediately occurred from the supercooled liquid, without any signs of a supercooled-liquid-to-glacial transformation.

Cyan line 3 in Figure 10 corresponds to irreversible crystallization into the metastable crystal*II polymorph upon heating, T*(*P*) = 236.7 K + 178.3·*P* K/GPa. The transition occurred either from the glacial state (see Figure 9c,d) or from the supercooled liquid state, bypassing the glacial state, depending on the thermal history. Cohen et al. [37] and Wiedersich et al. [18] reported a transition temperature of 237 K for the transition from the glacial state to the crystalline phase at atmospheric pressure. This is in good agreement with our transition temperatures measured at slightly lower rates than the 0.5 K/min heating rate employed by Wiedersich et al. [18]. Crystal*II remains (meta)stable upon subsequent cooling and heating up to line 4; that is, the region bounded by lines 3 and 4 is the region of existence of metastable crystal*II upon continuous heating from either the glacial or the glass state.

Gray line 4 describes the irreversible transition from crystal*II to crystal II upon continuous heating at about 0.3 K/min, *T*_II_(*P*) = 275 K + 155·*P* K/GPa; Figure 9d (run 14a) shows an example of the transition at 0.48 GPa. Baran et al. [32] established polymorphism in TPP by differential scanning calorimeter studies of TPP at atmospheric pressure. They showed that a new polymorph is obtained directly from the liquid phase by cooling from room temperature down to 245 K and reheating after 15 min dwell time at 245 K; upon heating, crystallization of the new polymorph occurred at 270 K. This temperature agrees reasonably well with the value of line 4 at atmospheric pressure. The P–T region of the phase diagram between lines 4 and 5 indicates the region of existence of metastable crystal II. Upon further heating, metastable crystal II irreversibly transforms into the stable equilibrium crystal I at temperatures indicated by the olive line 5, *T*_I_(*P*) = 286 K + 163·*P* K/GPa. Figure 9d shows such a transformation at *P* = 0.48 GPa (run 14a). This transition behavior is different from that at atmospheric pressure, at which the metastable polymorph melted directly at *T*_m_ = 291.6 K without transformation to the stable crystal I [32]. Transitions through lines 3, 4, and 5 are transitions of the first order, as indicated by jumps or kinks in *κ*(*T*) (see, for example, Figure 9d). Accordingly, one phase passes into another as a whole, without forming a mixture of crystalline phases.

Red line 6 in Figure 10 shows the melting conditions of TPP, *T*_m_(*P*) = 298 K + 178·*P* K/GPa. At atmospheric pressure, Mizukami et al. obtained *T*_m_ = 297.8 ± 0.2 K from their heat-capacity data [16], whereas DSC data yielded *T*_m_ = 299.1 K [32]; both results are in good agreement with our data.

## 3. Discussion

The results of both the temperature and pressure dependence of *κ* of the amorphous glass and glacial states and of the crystalline phases of TPP provide a new possibility to evaluate the theory of two-channel heat transfer by phonons and diffusons and the recently published unified theory of thermal transport in crystals and glasses [38]. We note that the magnitude and pressure dependence of *B*(*P*) in Equation (5) for the crystal phases and the corresponding results for *κ*_0_(*P*) in Equation (2) for the glass and glacial states are of similar sizes. This is a key finding which suggests that the same mechanism determines the *κ*(*T*, *P*) of glass and glacial states and the contribution of diffusons *B*(*T*,*P*) to the *κ* of crystals.

In the unified theory [38], the *κ* of crystals and glasses is described as the sum of two independent contributions: the first is the standard Peierls contribution to the *κ*, which is associated with particle-like propagation of phonon wave packets (Eucken law), and the second is a ‘coherences’ contribution, related to wave-like tunneling phenomena. In the case of *κ* of the glass and glacial states of TPP, the standard Peierls contribution to *κ* is absent or negligible, and the *κ* is described only by the second term, which is written as Equation (1). In this case, the activation energy, *E*, is the energy of the dominant excitations, which mainly determine the heat transfer channel associated with tunneling. In the high-temperature limit, when *E << T*, expressed in K, exp(−*E/T*) → 1, the *κ* of the glass and glacial states ceases to depend on temperature and reaches its maximum value, *κ*_0_(*P*), which is roughly the contribution of diffusons *B*(*P*) in crystals. The theoretical basis for this deduction is as follows.

In dielectric solids, two types of collective excitations contribute additively in two-channel heat transfer, propagons, *κ_p_* (*T*), and diffusons *κ_diff_* (*T*), in the terminology of Allen et al. [5]:

*κ* (*T*) = *κ_p_* (*T*) + *κ_diff_* (*T*). (6)

Propagons are identical to acoustic phonons in ordered crystalline materials and are considered to be low-frequency excitations of the phonon type in disordered solids.

In the general case, the contribution of propagons to *κ* determines the entire ensemble of propagons, as quantum excitations of the boson type with a density of vibration states, *g*(*ω*):

(7) $$\kappa_{p}\left(T\right)=\int_{0}^{\omega_{max}}g\left(\omega \right)C\left(\frac{\omega }{T}\right)D_{p}\left(\omega ,T\right)d\omega ,$$

where *ω* is phonon angular frequency, *C* (*ω*/*T*) is the spectral volumetric heat capacity. In the harmonic approximation, it depends on the relationship between frequency and temperature:

(8) $$C\left(\frac{\omega }{T}\right)=k_{B}{\left[\frac{\hbar \omega /2k_{B}T}{\sinh(\hbar \omega /2k_{B}T)}\right]}^{2}.$$

Low-frequency propagons have a large diffusivity *D_p_* (*ω*, *T*), which rapidly decreases with increasing excitation frequency; in the case of crystals, their intensity also decreases with increasing temperature due to an increase in the intensity of Umklapp processes as a result of three-phonon interactions (*U*—processes). This factor determines various high-temperature approximations of *κ_p_* (*T*); crystal-like for ordered and glass-like for disordered solids (see Table 5).

A new channel of thermal conductivity, *κ_diff_* (*T*), was proposed in Refs. [3,5] and developed in refs. [38,39]; it is realized by means of diffusons, and is the same contribution as the ‘coherences’ contributions, which is related to the wave-like tunneling and loss of coherence between different vibrational eigenstates [38]. It follows that the temperature dependence of *κ_diff_* has the same functional dependence; it can be approximated by using the minimum thermal conductivity model [35,40,41] or calculated theoretically, using complex computer calculations of the density of vibrational states, *g* (*ω*), and the diffusivity, *D_diff_* (*ω*), of diffusons for some substances [41,42,43,44,45,46,47,48,49,50,51,52,53,54,55,56].

In the general case, the diffuson contribution to *κ* is determined by the entire ensemble of diffusons as quantum excitations of the boson type with a density of frequency state (DOS) *g* (*ω*):

(9) $$\kappa_{diff}\left(T\right)=\int_{0}^{\omega_{max}}g(\omega )C\left(\omega /T\right)D_{diff}\left(\omega \right)d\omega .$$

The diffusivity, *D_diff_* (*ω*), of this thermal conductivity component is determined by interactions between closely spaced branches in the dispersion spectrum for both ordered and disordered solids. A characteristic feature of this function is that it decreases inversely with frequency. This feature, combined with the Debye model assumption that DOS *g* (*ω*)~*ω*^2^, is fundamental to the minimum thermal conductivity model [35]. The thermal conductivity due to one longitudinal and two transverse acoustic excitations can be written as the sum of three integrals:

(10) κminT= π61/3kBn2/3∑iviTΘi2∫0ΘiTx3ex−12dx.

The model of minimum thermal conductivity, *κ_min_*(*T*), qualitatively describes the increase in *κ* with increasing temperature and well predicts the saturation of the *κ_min_*(*T*) curve. The exponential dependence of *κ_diff_* (*T*) that was observed in this work is possibly due to the fact that the diffuson thermal conductivity, *κ_diff_* (*T*), is determined by the diffusivity of diffusons of dominant low-energy excitations, *D_diff_* (*ω*)~*ω*^−1^, in the frequency range where the ratio *g* (*ω*)/*D_diff_* (*ω*) becomes maximum. The average energy of such excitations is approximately equal to the value of the parameter *E* expressed in K of the exponential approximation of the dependence *κ_diff_* (*T*) = *κ_0_*(*T*) exp(−*E/T*). We note that such an approximation is the result of the universal behavior of both the real DOS *g* (*ω*), taking into account its characteristic feature (the boson peak in *g* (*ω*)/*ω*^2^), and the approximation *D_diff_* (*ω*)~*ω*^−1^.

To summarize the main features of *κ* of TPP above 100 K, we note that the temperature dependence in the crystalline phases differs sharply from that in the glass and glacial states. In particular, the *κ* of the crystalline phases decreases with temperature, whereas that of the glass and glacial states increases or remains constant. The decreasing *κ*(*T*) of the crystalline phases is typical for heat conduction by phonons, which are mainly scattered in three phonon Umklapp processes. The extent of phonon heat conduction (and phonon–phonon scattering) is reflected in the size of *A* in Equation (3). However, the simplistic model of *κ*(*T*) of the crystalline phases also requires a temperature-independent contribution, *B*. The magnitude of *B* differs between the crystal phases, and an increasing *B* is associated with a change in the crystal structure during polymorphic transformations, namely with the number of molecules in the unit cell [9,10,11]. As shown here, the *κ*(*T*) of fully amorphous states, such as that of the glass, is described well by the term associated with heat conduction of diffusons only, and in the case of crystals, *κ*(*T*) is associated with the two-channel heat transfer by phonons and diffusons. The observation that the diffusons’ contribution shows the same pressure dependence in crystalline phases as in amorphous phases indicates that the same mechanism is responsible for this channel of heat transfer in crystals and amorphous states.

## 4. Materials and Methods

Triphenyl phosphite was supplied by Fisher Scientific. The material, with a stated purity better than 99 %, was used without further purification. The molecule of triphenyl phosphite P(OC_6_H_5_)_3_ is highly flexible, and as many as six torsion angles can be identified. Triphenyl phosphite has a glass transition at (200 ± 2) K, and the stable crystalline phase melts at 299.1 K at 1 atm; it has a trigonal lattice with the hexagonal space group R3 (Z = 18) and one molecule in the asymmetric unit [57,58]. The lattice parameters at 200 K were determined to be a = 37.887(1) Å and c = 5.7567(2) Å (V = 7156(1) Å^3^). Another monoclinic polymorphic modification of triphenyl phosphite, which crystallizes in the P21/n space group with one independent molecule in the unit cell [59], melts at 291.6 K.

**Figure 11:**
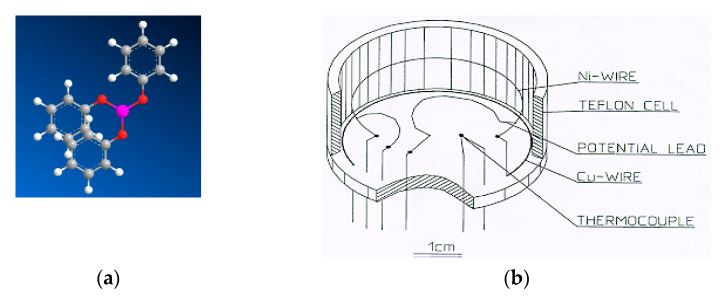
Illustration of the chemical structure of triphenyl phosphite and the custom-made sample cell with hot-wire: (**a**) the chemical structure of a triphenyl phosphite molecule (P(OC_6_H_5_)_3_; (**b**) Illustration of the custom-made sample cell with hot-wire.

The thermal conductivity, *κ*, was measured under pressure, using the transition hot-wire method [60,61]. Figure 11 shows the chemical structure of the triphenyl phosphite molecule and the custom-made sample cell with hot-wire used for the measurements of *κ* triphenyl phosphite.

The hot-wire probe was a 40 mm long Ni-wire that was mounted in a Teflon sample cell. The cell was thereafter filled with the sample and sealed with a Teflon lid before being inserted in a piston-cylinder device of 45 mm internal diameter. A 200-ton hydraulic press supplied the load, and the pressure was kept constant to within ±0.5 MPa during isobaric measurements by a proportional–integral–derivative controller (Eurotherm 2408). The pressure was determined from the load by using an empirical correction for friction, which was established in a separate experiment, using the pressure dependence of a manganin resistance; the inaccuracy in pressure is estimated to be 40 MPa at 1 GPa. The temperature was varied by heating or cooling the whole piston-cylinder device, using an external electric heater or liquid nitrogen; it was measured by a Chromel–Alumel thermocouple placed inside the cell, with an inaccuracy of ±0.5 K. The maximum cooling and heating rates were 2 K/min and 0.5 K/min, respectively. The results on heating and cooling differ slightly due to the reversal of frictional forces between the piston (and cell) and the cylinder, and changes in thermal gradients. Due to the relatively fast cooling rate employed here, these effects caused differences in *κ* of up to 1–2% between results on cooling and heating.

In each measurement of *κ*, the hot-wire probe is heated by 3–4 K by a 1.4 s long current pulse of nominally constant power. Its temperature rise versus time is determined by 29 electrical resistance measurements during the pulse, which are transformed to temperatures via the resistance–temperature relation of the Ni-wire. Subsequently, the value of *κ* is obtained by fitting the analytical solution for the temperature rise versus time to the measured values with an estimated inaccuracy of ±2%.

The thermal conductivity typically changes discontinuously between different phases, and, because of the transition enthalpy, the method gives anomalous values for *κ* in phase-transition ranges. This makes it straightforward to detect phase transitions and determine the stability ranges of phases. Moreover, an artificial peak arises in *κ* in glass-transition ranges due to the time dependence of the heat capacity. This feature has been analyzed and discussed in detail: the peak is superimposed on the (real) change in the slope of *κ*, which is caused by the increase in the thermal expansion coefficient at the glass transition temperature [36]. The most significant results of the analysis are that the anomalous peak maximum in *κ* occurs at a relaxation time of 0.3 s, and the peak is observed in the approximate relaxation time range of 10^−3^–10^3^ s.

## 5. Conclusions

This work provides a comprehensive study and analysis of the thermal conductivity of the various phases and states of solid triphenyl phosphite at a pressure up to 0.5 GPa for temperatures in the 90–380 K range. In particular, we analyzed the heat transfer processes in the stable and metastable crystalline phases and in the amorphous glass and glacial states. Both the magnitude and temperature dependence of *κ* change at the transitions between the states and phases, and this is due to changes in structural order and crystal structure; the transition coordinates provide a transitional pressure–temperature diagram of the states of triphenyl phosphite, which is presented. We find three crystalline phases, designated as phase I, II, and *II, where the thermal conductivity behavior suggests that phase *II is a poorly crystalline version of the metastable phase II. The numerous phases and states of triphenyl phosphite, as well as the results for both the pressure and temperature dependence of the thermal conductivity, provide good possibilities to evaluate theory for thermal conductivity. The thermal conductivity of the glass and glacial states is described well by a term associated with the heat conduction of diffusons only, which is one of the heat-transfer channels of the two-channel heat-transfer theory of dielectric solids; the thermal conductivity can be represented by an Arrhenius-type function: *κ*(*T*, *P*) = *κ*_0_(*P*) exp(−*E*/*T*), where *E* is the activation energy expressed in K, and *κ*_0_ is a pre-exponential factor, which varies linearly with pressure. In the case of the crystalline phases, the thermal conductivity is associated with two-channel heat transfer by phonons and diffusons, and it is well described by the expression *κ*(*T*) = *A*/*T* + *B*, where *A*/*T* is the contribution from propagating phonons, and the second term, *B*, is the contribution associated with diffusons. We find that the contribution of diffusons B in the crystalline phases, where phonon–phonon scattering processes predominate, shows the same pressure dependence as that in the amorphous phases, suggesting that the contributions have the same origin. However, this study appears to be the first that provides such a comparison; a systematic comparison of many crystalline and amorphous solids is required before we can conclude that this result is universal. The results for the thermal conductivity of triphenyl phosphite are well-described within the framework of the unified theory of two-channel heat transfer in dielectric solids, including both complex and simple crystalline phases and amorphous states [38,39].

## Figures and Tables

**Table 1:** Parameters *E* (expressed in K) and *κ*_0_ of the fitted function, Equation (1), of the glass state. Δ*T* is the temperature interval of the experimental data. The right column shows the conditions for obtaining the glass and for measurements of its *κ*.

*P*, GPa	run	*κ*_0_, Wm^−1^K^−1^	*E*, K	Δ*T*, K	Experimental Conditions
0	43	0.149	14	166–206	Cooling from liquid (to glass) at −1.7 K/min. Measurements of *κ* on heating at 0.46 K/min.
0.05	03	0.148	11.1	175–96	Cooling from liquid at −2 K/min, and (simultaneous) measurements of *κ* on cooling.
0.05	04	0.153	13.6	98–210	Continued run 03, measurements of *κ* on heating at 0.3 K/min.
0.05	18	0.153	13.8	183–203	Cooling from liquid at −1.6 K/min, measurements of *κ* on heating at 0.4 K/min
0.10	36	0.166	25	190–214	Cooling from liquid −1.3 K/min, measurements of *κ* on heating at 0.58 K/min.
0.15	27a	0.16	11.5	204–225	Cooling from liquid at −1.6 K/min, measurements of *κ* on heating at 0.4 K/min.
0.48	10	0.196	10	237–89	Cooling from liquid at −2.0 K/min, measurements of *κ* on cooling.
0.48	11	0.204	14.6	89–267	Continued run 10, measurements on heating at 0.08 K/min.

**Table 2:** Parameters *E* (expressed in K) and *κ*_0_ of the fitted function, Equation (1), of the glacial state: Δ*T* is the temperature interval of the experimental data. The right column shows the conditions of the measurements.

*P*, GPa	run	*κ*_0_, Wm^−1^K^−1^	*E*, K	Δ*T*, K	Experimental Conditions
0	110	0.137	12.0	169–126	Cooling
0	110	0.141	16.6	128–204	Heating
0	120	0.149	8.8	187–133	Cooling
0	120	0.157	15.5	133–197	Heating
0.05	20	0.152	7.0	191–100	Cooling
0.05	21	0.16	11.5	98–182	Heating
0.10	107	0.167	7.0	217–98	Cooling
0.10	108	0.174	12.0	97–213	Heating
0.10	59	0.156	8.0	223–159	Cooling
0.10	60	0.169	21.0	156–227	Continued run 59, heating
0.10	61	0.154	6.0	160–100	Cooling
0.10	62a	0.167	15.4	100–250	Continued run 60, heating
0.48	13	0.212	13.3	297–89	Cooling
0.48	14a	0.214	14.1	87–260	Continued run 13, heating
0.49	103	0.199	9.7	255–105	Cooling
0.49	104	0.21	15.4	106–260	Continued run 103, heating
0.5	39a	0.199	18.1	235–171	Cooling
0.5	39b	0.204	21.0	171–230	Continued run 39a, heating
0.5	72	0.193	14.0	236–82	Cooling
0.5	73	0.2	17.3	82–236	Continued run 72, heating
0.5	75	0.201	17.0	220–260	Cooling
0.5	76	0.211	28.0	220–272	Continued run 75, heating
0.5	79	0.202	14.5	270–198	Cooling
0.5	80	0.207	19.0	200–256	Continued run 79, heating
0.5	81	0.205	17.0	277–222	Continued run 80, cooling
0.5	82	0.213	25.0	222–270	Continued run 81, heating
0.5	94a	0.196	11.0	270–105	Cooling
0.5	94b	0.203	14.5	105–230	Heating after cooling
0.5	96	0.215	14.0	160–274	Heating

**Table 3:** Thermal conductivity contribution of propagating phonons, *A*/*T*, and diffusons, *B*, in Equation (3), which describes the thermal conductivity of TPP crystals within ±1.5% in the specified temperature interval. The right column shows the conditions for measurements of *κ*(*T*).

*P*, GPa	run	*A*, Wm^−1^	*B*, Wm^−1^K^−1^	Temperature Interval, K	Experimental Conditions
Crystal I					
0.05	23a	26.8	0.129	296–106	Cooling, −1.4 K/min
0.05	23b	28.8	0.127	106–290	Heating, 0.5 K/min
0.48	14b	34.2	0.185	380–306	Cooling, −0.15 K/min
Crystal II					
0	42a	12.5	0.13	273–197	Cooling, −1.4 K/min,
0	42b	12.5	0.13	197–270	Continued run 42a; heating, 0.4 K/min
0	43	10.5	0.13	231–255	Heating, 0.46 K/min
0.45	08c	16.3	0.21	357–330	Cooling, −0.2 K/min
0.47	50e	15.3	0.218	300–200	Cooling
0.47	51a	15.2	0.22	183–348	Heating
0.47	51b	14	0.22	351–298	Cooling
Crystal*II					
0.05	05	2.76	0.17	260–102	Cooling, −1.4 K/min
0.05	06a	3.94	0.165	100–270	continued run 05; heating, 0.2 K/min
0.10	62b	0	0.182	264–286	Heating, 0.4 K/min
0.15	27b	0	0.192	265–299	Heating, 0.4 K/min
0.3	32	0	0.213	296–317	Heating, 0.37 K/min
0.45	08b	0	0.254	297–356	Heating, 0.46 K/min
0.47	48	3.3	0. 22	267–116	Cooling, −1.6 K/min
0.47	49a	1.7	0.232	113–322	Continued run 48; heating
0.47	49b	5.2	0.227	322–296	Continued run 49a; cooling
0.47	50a	0	0.245	297–323	Continued run 49b; heating
0.47	50b	8	0.227	341–297	Cooling
0.47	50c	3.3	0.243	297–356	Heating

**Table 4:** Values of *A*_av_ and *dA*_av_/*dP* in Equation (4), *B*_av_ and *dB*_av_/*dP* in Equation (5), and *a* and *b* in Equation (2) for the crystal phases I, II, and *II, and the glass and glacial states.

	*A*_av_, Wm^−1^	*dA*_av_/*dP*, Wm^−1^GPa^−1^	*B*_av_, Wm^−1^K^−1^	*dB*_av_/*dP*, Wm^−1^K^−1^GPa^−1^	*a*, Wm^−1^K^−1^	*b*, Wm^−1^K^−1^GPa^−1^
crystal I	27	14.9	0.121	0.132		
crystal II	11.9	7.17	0.13	0.187		
crystal*II	1.27	3.28	0.164	0.153		
glacial	-	-			0.147 (cooling) 0.157 (heating)	0.1 0.1
glass	-	-			0.148	0.113

**Table 5:** High-temperature approximations of contributions to thermal conductivity for two-channel heat transfer by propagons, *κ_p_* (*T*), and by diffusons, *κ_diff_* (*T*). *D_p_* (*ω, T*) is diffusivity of propagons, and *D_diff_* (*ω*) is the diffusivity of diffusons in the case of crystal-like behavior and glass-like behavior of thermal conductivity of solids, respectively.

Contributions	Glass-like Behavior	Crystal-like Behavior
*D_p_* (*ω, T*)	*ω* ^−4^	*T* ^−1^ *ω* ^−1^
*D_diff_* (*ω*)	*ω* ^−1^	*ω* ^−1^
*κ_p_* (*T*), high-temperature limit	*T* ^0^	*T* ^−1^
*κ_diff_* (*T*), high-temperature limit	exp(*−E/T*)	exp(*−E/T*)

## Data Availability

The data presented in this study are available on request from the corresponding authors.

## References

[1] Berman R. (1976). Thermal Conduction in Solids.

[2] Konstantinov V.A., Manzhelii V.G., Strzhemechny M.A., Smirnov S.A. (1988). The Λ∝ 1/T law and isochoric thermal conductivity of rare gas crystals. Sov. J. Low Temp. Phys..

[3] Allen P.B., Feldman J.L. (1993). Thermal conductivity of disordered harmonic solids. Phys. Rev. B.

[4] Feldman J.L., Kluge M.D., Allen P.B., Wooten F. (1993). Thermal conductivity and localization in glasses: Numerical study of a model of amorphous silicon. Phys. Rev. B.

[5] Allen P.B., Feldman J.L., Fabian J., Wooten F. (1999). Diffusons, locons and propagons: Character of atomie yibrations in amorphous Si. Philos. Mag. B.

[6] McGaughey A.J.H., Kaviany M. (2004). Thermal conductivity decomposition and analysis using molecular dynamics simulations. Part I. Lennard-Jones argon. Int. J. Heat Mass Transf..

[7] Krivchikov A.I., Sharapova I.V., Korolyuk O.A., Romantsova O.O., Bermejo F.J. (2009). Experimental evidence of the role of quasilocalized phonons in the thermal conductivity of simple alcohols in orientationally ordered crystalline phases. Low Temp. Phys..

[8] Korolyuk O.A. (2011). Thermal conductivity of molecular crystals of monatomic alcohols: From methanol to butanol. Low Temp. Phys..

[9] Krivchikov A.I., Romantsova O.O., Korolyuk O.A., Vdovichenko G.A., Horbatenko Y.V. (2015). Specific features of heat transfer in the orientationally ordered phases of molecular crystals in the region with predominant phonon-phonon scattering. Low Temp. Phys..

[10] Krivchikov A.I., Korolyuk O., Sharapova I.V., Tamarit J.L., Bermejo F.J., Pardo L.C., Rovira-Esteva M., Ruiz-Martin M.D., Jezowski A., Baran J. (2012). Effects of internal molecular degrees of freedom on the thermal conductivity of some glasses and disordered crystals. Phys. Rev. B.

[11] Romantsova O.O., Horbatenko Y.V., Krivchikov A.I., Korolyuk O.A., Vdovichenko G.A., Zloba D.I., Pyshkin O.S. (2017). Anomalous heat transfer in two polymorphs of para-bromobenzophenone. Low Temp. Phys..

[12] Krivchikov A.I., Jeżowski A., Ramos M.A. (2022). Thermal conductivity of glasses and disordered crystals. Low-Temperature Thermal and Vibrational Properties of Disordered Solids: A Half-Century of Universal “Anomalies” of Glasses.

[13] Babkov L.M., Baran J., Davydova N.A., Ivlieva I.V., Ponezha E.A., Reznichenko V.Y. (2016). Infrared Spectra of Triphenyl Phosphite and Their Interpretation on the Basis of Quantum Chemistry Calculation. Ukr. J. Phys..

[14] Ha A., Cohen I., Zhao X., Lee M., Kivelson D. (1996). Supercooled Liquids and Polyamorphism. J. Phys. Chem..

[15] van Miltenburg K., Blok K. (1996). Calorimetric Investigation of a New Solid Phase in Triphenylphosphite. J. Phys. Chem..

[16] Mizukami M., Kobashi K., Hanaya M., Oguni M. (1999). Presence of Two Freezing-In Processes Concerning α-Glass Transition in the New Liquid Phase of Triphenyl Phosphite and Its Consistency with “Cluster Structure” and “Intracluster Rearrangement for α Process” Models. J. Phys. Chem. B.

[17] Krivchikov A.I., Andersson O. (2016). Thermal Conductivity of Triphenyl Phosphite’s Liquid, Glassy, and Glacial States. J. Phys. Chem. B.

[18] Wiedersich J., Kudlik A., Gottwald J., Benini G., Roggatz I., Rössler E. (1997). On Polyamorphism of Triphenyl Phosphite. J. Phys. Chem. B.

[19] Johari G.P., Ferrari C. (1997). Calorimetric and Dielectric Investigations of the Phase Transformations and Glass Transition of Triphenyl Phosphite. J. Phys. Chem. B.

[20] Tarnacka M., Madejczyk O., Dulski M., Maksym P., Kaminski K., Paluch M. (2017). Is There a Liquid–Liquid Phase Transition in Confined Triphenyl Phosphite?. J. Phys. Chem. C.

[21] Kobayashi M., Tanaka H. (2016). The reversibility and first-order nature of liquid–liquid transition in a molecular liquid. Nat. Commun..

[22] Demirjian B.G., Dosseh G., Chauty A., Ferrer M.L., Morineau D., Lawrence C., Takeda A.K., Kivelson D., Brown S. (2001). Metastable Solid Phase at the Crystalline-Amorphous Border: The Glacial Phase of Triphenyl Phosphite. J. Phys. Chem. B.

[23] Lefort R., Hédoux A., Guinet Y., Cochin E., Descamps M. (2002). Fast intramolecular dynamics of triphenyl phosphite investigated by 2H NM. Eur. Phys. J. B.

[24] Senker J., Sehnert J., Correll S. (2004). Microscopic Description of the Polyamorphic Phases of Triphenyl Phosphite by Means of Multidimensional Solid-State NMR Spectroscopy. J. Am. Chem. Soc..

[25] Mierzwa M., Paluch M., Rzoska S.J., Zioło J. (2008). The Liquid−Glass and Liquid−Liquid Transitions of TPP at Elevated Pressure. J. Phys. Chem. B.

[26] Hédoux A., Guinet Y., Derollez P., Hernandez O., Paccou L., Descamps M. (2006). Micro-structural investigations in the glacial state of triphenyl phosphite. J. Non-Cryst. Solids.

[27] Mei Q., Siewenie J.E., Benmore C.J., Ghalsasi P., Yarger J.L. (2006). Orientational Correlations in the Glacial State of Triphenyl Phosphite. J. Phys. Chem. B.

[28] Hédoux A., Guinet Y., Descamps M. (2001). Size dependence of the Raman spectra in an amorphous-nanocrystalline mixed phase: The glacial state of triphenyl phosphite. J. Raman Spectrosc..

[29] Hedoux A., Dore J., Guinet Y., Bellissent-Funel M.C., Prevost D., Descamps M., Grandjean D. (2002). Analysis of the local order in the glacial state of triphenyl phosphite by neutron diffraction. Phys. Chem. Chem. Phys..

[30] Tanaka H. (2020). Liquid–liquid transition and polyamorphism. J. Chem. Phys..

[31] Hédoux A., Guinet Y., Derollez P., Hernandez O., Lefort R., Descamps M. (2004). A contribution to the understanding of the polyamorphism situation in triphenyl phosphite. Phys. Chem. Chem. Phys..

[32] Baran J., Davydova N.A., Drozd M. (2014). Polymorphism of triphenyl phosphite. J. Chem. Phys..

[33] Baran J., Davydova N.A., Drozd M., Krivchikov A. (2020). Effect of the clay nanomaterial laponite on the crystallization characteristics of nanocomposites TPP/laponite. Mol. Cryst. Liq. Cryst..

[34] Andersson S.P., Ross R.G. (1994). Thermal conductivity and heat capacity per unit volume of poly(methyl methacrylate) under high pressure. Int. J. Thermophys..

[35] Cahill D.G., Watson S.K., Pohl R.O. (1992). Lower limit to the thermal conductivity of disordered crystals. Phys. Rev. B.

[36] Andersson O. (1997). Simulation of a glass transition in a hot-wire experiment using time-dependent heat capacity. Int. J. Thermophys..

[37] Cohen I., Ha A., Zhao X., Lee M., Fischer T., Strouse M.J., Kivelson D. (1996). A Low-Temperature Amorphous Phase in a Fragile Glass-Forming Substance. J. Phys. Chem..

[38] Simoncelli M., Marzari N., Mauri F. (2019). Unified theory of thermal transport in crystals and glasses. Nat. Phys..

[39] Isaeva L., Barbalinardo G., Donadio D., Baroni S. (2019). Modeling heat transport in crystals and glasses from a unified lattice-dynamical approach. Nat. Commun..

[40] Huang M., Liu X., Zhang P., Qian X., Feng Y., Li Z., Pan W., Wan C. (2021). Thermal conductivity modeling on highly disordered crystalline Y_1−*x*_Nb*_x_*O_1.5+*x*_: Beyond the phonon scenario. Appl. Phys. Lett..

[41] Kumar G., Van Gessel F.G., Elton D.C., Chung P.W. (2019). Phonon Lifetimes and Thermal Conductivity of the Molecular Crystal α-RDX. MRS Adv..

[42] Larkin J.M., McGaughey A.J.H. (2014). Thermal conductivity accumulation in amorphous silica and amorphous silicon. Phys. Rev. B.

[43] Cheng Z., Weidenbach A., Feng T., Tellekamp M.B., Howard S., Wahila M.J., Zivasatienraj B., Foley B., Pantelides S.T., Piper L.F.J. (2019). Diffuson-driven ultralow thermal conductivity in amorphous Nb_2_O_5_ thin films. Phys. Rev. Mater..

[44] Chen X., Weathers A., Carrete J., Mukhopadhyay S., Delaire O., Stewart D., Mingo N., Girard S.N., Ma J., Abernathy D. (2015). Twisting phonons in complex crystals with quasi-one-dimensional substructures. Nat. Commun..

[45] Hanus R., George J., Wood M., Bonkowski A., Cheng Y., Abernathy D.L., Manley M.E., Hautier G., Snyder G.J., Hermann R.P. (2021). Uncovering design principles for amorphous-like heat conduction using two-channel lattice dynamics. Mater. Today Phys..

[46] Hanus R., Gurunathan R., Lindsay L., Agne M.T., Shi J., Graham S., Snyder G.J. (2021). Thermal transport in defective and disordered materials. Appl. Phys. Rev..

[47] Luo Y., Yang X., Feng T., Wang J., Ruan X. (2020). Vibrational hierarchy leads to dual-phonon transport in low thermal conductivity crystals. Nat. Commun..

[48] Zhou Y. (2021). Assessing the quantum effect in classical thermal conductivity of amorphous silicon. J. Appl. Phys..

[49] Lundgren N.W., Barbalinardo G., Donadio D. (2021). Mode localization and suppressed heat transport in amorphous alloys. Phys. Rev. B.

[50] Ohnishi M., Tadano T., Tsuneyuki S., Shiomi J. (2021). Strong Phonon Anharmonicity of Clathrate Compound at High Temperature. arXiv.

[51] Aryana K., Stewart D.A., Gaskins J.T., Nag J., Read J.C., Olson D.H., Grobis M.K., Hopkins P.E. (2021). Tuning network topology and vibrational mode localization to achieve ultralow thermal conductivity in amorphous chalcogenides. Nat. Commun..

[52] Braun J.L., King S.W., Hoglund E.R., Gharacheh M.A., Scott E.A., Giri A., Tomko J.A., Gaskins J.T., Al-Kukhun A., Bhattarai G. (2021). Hydrogen effects on the thermal conductivity of delocalized vibrational modes in amorphous silicon nitride (a−SiN_x_:H). Phys. Rev. Mater..

[53] Qian X., Zhou J., Chen G. (2021). Phonon-engineered extreme thermal conductivity materials. Nat. Mater..

[54] Zhang Z., Guo Y., Bescond M., Chen J., Nomura M., Volz S. (2022). Heat Conduction Theory Including Phonon Coherence. Phys. Rev. Lett..

[55] Caldarelli G., Simoncelli M., Marzari N., Mauri F., Benfatto L. (2022). A many-body Green’s function approach to lattice thermal transport. arXiv.

[56] Bernges T., Hanus R., Wankmiller B., Imasato K., Lin S., Ghidiu M., Gerlitz M., Peterlechner M., Graham S., Hautier G. (2022). Considering the Role of Ion Transport in Diffuson-Dominated Thermal Conductivity. Adv. Energy Mater..

[57] Hernandez O., Hédoux A., Lefebvre J., Guinet Y., Descamps M., Papoular R., Masson O. (2002). *Ab initio* structure determination of triphenyl phosphite by powder synchrotron X-ray diffraction. J. Appl. Crystallogr..

[58] Senker J., Lüdecke J. (2001). Structure Determination for the Crystalline Phase of Triphenyl Phosphite by Means of Multi-Dimensional Solid-State NMR and X-ray Diffraction. Z. Für Nat. B.

[59] Golovanov D.G., Lyssenko K.A., Antipin M.Y., Vygodskii Y.S., Lozinskaya E.I., Shaplov A.S. (2005). Long-awaited polymorphic modification of triphenyl phosphite. CrystEngComm.

[60] Håkansson B., Andersson P., Bäckström G. (1988). Improved hot-wire procedure for thermophysical measurements under pressure. Rev. Sci. Instrum..

[61] Andersson O., Inaba A. (2005). Thermal conductivity of crystalline and amorphous ices and its implications on amorphization and glassy water. Phys. Chem. Chem. Phys..

